# Book Reviews

**Published:** 1905-06

**Authors:** 


### BOOK REVIEWS.

---

LE DANGER DE LA MORTE APPARENTE SUR LES CHAMPS DE BATAILLE. Par Le Docteur Icard (De Marseille). A. Maloine, Editor. 25-27 Rue de l'Ecole de Medicine, Paris.

The fear of premature burial is a legitimate one and is fully justified by observed facts. The danger of apparent death is to be feared under all circumstances, but more especially during times of epidemics and of war when hasty interment greatly augment the possibilities of error. Dr. Icard has made a special study of these questions. In a previous volume he has considered the danger of apparent death in time of epidemics; the present work is concerned with the danger of apparent death on the field of battle. There is no work upon this subject which seems to merit extended study, and the author has sought to supply the deficiency. The appearance of the book is especially appropriate at the present time when the Russo-Japanese war offers opportunities of applying its suggestion. The author proposes as an infallible test the hypodermatic injection of a solution of fluorescein. If the blood is still circulating the substance is absorbed and the body rapidly turns an intense yellow, while the eyeballs become of an emerald green. The test can be made very rapidly, and the appearances are so marked as to render it impossible not to attract attention.

---

HARRINGTON'S PRACTICAL HYGIENE. A Treatise on Hygiene and Sanitation. For Students, Practitioners, Health Officers, etc. By Charles Harrington, M.D., Assistant Professor of Hygiene in Harvard University Medical School, Boston. New (3d) edition, thoroughly revised. In one octavo volume of 793 pages, with 118 engravings and 12 plates. Cloth, $4.25, *net*. Lea Brothers & Co., Publishers, Philadelpia and New York, 1905.

No department in the realm of medicine approaches in importance Hygiene and Sanitation—the science and art of conserving the health, energy, wealth and welfare of the individual and of the community. An authoritative book, covering the entire subject clearly and comprehensively, is therefore an essential to the full execution of professional responsibilities. Dr. Harrington's work was accepted as the authority upon the appearance of its first edition. He treats his subject broadly and with careful attention to details, his purpose being to furnish a clear, complete, well-illustrated volume equally adapted to the needs of the student, practitioner and health officer. The success of the work is well shown in the demand which has exhausted two large editions in less than four years. As the succeeding editions are called for, the author by careful revision, elision of obsolete matter and addition of new, keeps his work well abreast of the advances in a subject by no means stagnant. The new section on Infection, Susceptibility and Immunity will prove a valuable and interesting feature of the present edition. Evidences of searching revision will be found throughout the book, the alterations and additions necessitating a considerable increase in both text and illustrations, although the price remains at its previous very moderate figure.

---

### GRAY'S ANATOMY.

Messrs. Lea Brothers & Co. have pleasure in announcing a new edition of Gray's Anatomy, to be published about midsummer, and embodying nearly two years of labor on the part of the editor, J. Chalmers DaCosta, M.D., of Philadelphia, and a corps of special assistants.

Commensurately with the importance of the largest selling medical works ever published, this new edition will present a revision so thorough and searching that the entire book has been reset in new type. In addition to the changes necessary to bring it abreast of the most modern knowledge of its subject, several important alterations have been made with the view of adapting it still more closely to present-day teaching methods, and in fact to anticipate the trend of anatomical work and study.

Thus, while the older nomenclature is used, the new names (B.N.A.) follow in brackets; the section on Embryology and Histology at the back of the present "Gray" has been distributed throughout the new edition in the shape of embryological, histological and biological references and paragraphs bearing directly on the part under consideration, thus contributing to a better and easier understanding.

The illustrations have come in for their full share of the general revision, so that at this writing more than 400 new and elaborate engravings in black and colors have been prepared. "Gray" has always been noted for its richness of illustration, but the new edition far exceeds anything that has hitherto been attempted.

No medical text-book has ever approached "Gray" in sturdy longevity and accumulating strength. Notwithstanding the many would-be competitors which during nearly fifty years have periodically appeared and endeavored to share in its ever-increasing popularity, this wonderful creation of a genius who lived barely long enough to realize that his work was done—how well he never knew—goes on and on, each succeeding year bringing new friends and strengthening the fealty of the old.

The editor and publishers have spared neither labor nor expense to keep "Gray" at the forefront of anatomical knowledge, and there seems to be no reason to doubt that its next fifty years will pass as smoothly and as successfully as have those past.

---

M. Roux communicated to the Paris Académie des Sciences at a meeting on February 20th a note from M. Nicolle, director of the Pasteur Institute at Tunis, announcing that he had succeeded in inoculating two monkeys of the Macacus genus with leprosy. Sixty-two days after the inoculation well-marked leprous lesions were visible behind the ears.

---

In the State of Ohio there is a law forbidding the issue of marriage licenses to habitual drunkards. A similar measure is to be submitted to the New Jersey State Legislature.

